# A new model of forelimb ecomorphology for predicting the ancient habitats of fossil turtles

**DOI:** 10.1002/ece3.8345

**Published:** 2021-11-25

**Authors:** Thomas W. Dudgeon, Marissa C. H. Livius, Noel Alfonso, Stéphanie Tessier, Jordan C. Mallon

**Affiliations:** ^1^ Department of Ecology and Evolutionary Biology University of Toronto Toronto Ontario Canada; ^2^ Department of Natural History Royal Ontario Museum Toronto Ontario Canada; ^3^ Ottawa‐Carleton Geoscience Centre and Department of Earth Sciences Carleton University Ottawa Ontario Canada; ^4^ Beaty Centre for Species Discovery and Zoology Section Canadian Museum of Nature Ottawa Ontario Canada; ^5^ Beaty Centre for Species Discovery and Palaeobiology Section Canadian Museum of Nature Ottawa Ontario Canada

**Keywords:** ecomorphology, morphometrics, testudines

## Abstract

Various morphological proxies have been used to infer habitat preferences among fossil turtles and their early ancestors, but most are tightly linked to phylogeny, thereby minimizing their predictive power. One particularly widely used model incorporates linear measurements of the forelimb (humerus + ulna + manus), but in addition to the issue of phylogenetic correlation, it does not estimate the likelihood of habitat assignment. Here, we introduce a new model that uses intramanual measurements (digit III metacarpal + non‐ungual phalanges + ungual) to statistically estimate habitat likelihood and that has greater predictive strength than prior estimators. Application of the model supports the hypothesis that stem‐turtles were primarily terrestrial in nature and recovers the nanhsiungchelyid *Basilemys* (a fossil crown‐group turtle) as having lived primarily on land, despite some prior claims to the contrary.

## Graphical abstract

Various morphological proxies have been used to infer habitat preferences among fossil turtles and their early ancestors, but most are tightly linked to phylogeny, thereby minimizing their predictive power. Here, we introduce a new model that uses intramanual measurements (digit III metacarpal + non‐ungual phalanges + ungual) to statistically estimate habitat likelihood and that has greater predictive strength than prior estimators. Application of the model supports the hypothesis that stem turtles were primarily terrestrial in nature and recovers the nanhsiungchelyid Basilemys (a fossil crown‐group turtle) as having lived primarily on land, despite some prior claims to the contrary.

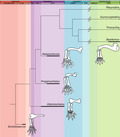

## 1 INTRODUCTION

Among the most persistent challenges in the history of evolutionary biology has been the question of the origin and evolution of turtles and their nearest ancestors. Fortunately, new fossil finds, reinterpretations of old ones, and molecular analyses have recently combined to provide a much clearer picture of the early evolution of the clade (recently summarized by Lyson & Bever, 2020). Whereas turtles were once variably attributed to “Anapsida,” to early branching diapsids, and to the sister taxon of Lepidosauria (among others), thanks to converging molecular phylogenetic analyses, they are now recognized as the extant sister group to archosaurs (Crawford et al., 2015; Field et al., 2014; Thomson et al., 2014). Whereas the primitive habitat (terrestrial vs. aquatic) of turtles was once a subject of debate, recent consensus maintains that the earliest stem‐turtles, such as *Eunotosaurus africanus* and *Pappochelys rosinae*, were fully terrestrial, and perhaps even fossorial (Joyce, 2015; Lyson & Bever, 2020).

Despite these advancements in understanding, there is dissent among turtle workers regarding the habits of many other fossil forms. For example, the Upper Triassic stem‐turtles *Odontochelys semitestacea*, *Proterochersis robustus*, and *Proganochelys quenstedtii* have variably been attributed both terrestrial and aquatic lifestyles (Benson et al., 2011; Joyce, 2015; Joyce & Gauthier, 2004; Li et al., 2008; Scheyer & Sander, 2007). Whether or not stem‐turtles exhibited “considerable ecological plasticity” (Benson et al., 2011:560), therefore, depends on which habitat assignments are accepted. Those assignments similarly impact the phylogenetic inference of the habitats of the first crown turtles (clade: Testudines). The habitats of still other fossil crown turtles, such as the Upper Cretaceous nanhsiungchelyids, have likewise been debated (recently summarized by Mallon & Brinkman, 2018). The persistent conflict stems largely from the plethora of ecomorphological proxies that have been applied to the question of fossil turtle habitats. These include analyses of shell proportions, bone isotopes, skull and middle ear morphology, osteohistology, and limb proportions (Benson et al., 2011; Dziomber et al., 2020; Foth et al., 2017, 2019; Joyce & Gauthier, 2004; Lichtig & Lucas, 2017; Rivera, 2008; Scheyer & Sander, 2007).

Of the above proxies, cranial shape and middle ear morphology individually correlate only weakly with habitat and perform poorly for fossil taxa when evaluated against external corroborators (e.g., sedimentary environments; Foth et al., 2017, 2019). Similarly, while 2‐ and 3‐dimensional shell shape has been widely used by researchers to infer paleohabitat (e.g., Benson et al., 2011; Dziomber et al., 2020; Rivera, 2008; Rivera et al., 2014), primarily because shells are readily available and easy to measure, its actual correlation with habitat is not particularly strong; turtle shells vary for many reasons unrelated to habitat preference (Dziomber et al., 2020). Butterfield et al. (2021) investigated the combined relationship between forelimb (bony and soft tissue), skull, and shell morphology with habitat and ecology, finding stronger relationships than previous studies, but their analysis lacks the broad taxonomic scale and large sample size needed to make predictions of fossil taxa. The most cited ecomorphological study to date is that of Joyce and Gauthier (2004), who plotted forelimb proportions (humerus + ulna + manus) on ternary diagrams to classify turtle habitat with some success. The strength of their approach lies in the fact that forelimb proportions are easy to measure, and the ecological signal is unlikely to be altered by taphonomic crushing (unlike shell doming). The method also applies to the earliest nontestudinate taxa, which did not possess fully formed shells (but see Joyce, 2015). The authors found that the turtle ecomorphs separate primarily by relative manus length, which can discriminate between fully terrestrial and fully aquatic forms, with taxa occupying intermediate habitats falling in between.

Despite the simplicity and relative predictive strength of Joyce and Gauthier's method, it nevertheless has its shortcomings. As originally outlined, the method is largely qualitative in nature, relying on visually assessing overlap in ecomorphospace. It is therefore difficult to quantify the likelihood of habitat assignments. The method also does not account for the effects of phylogeny, which Benson et al. (2011) showed is an even better predictor of forelimb proportions. Nor does the method account for variation within the manus, including the relative lengths of the digits and their unguals. The last source of variation is worth exploring, because Joyce and Gauthier (2004) showed that relative manus length is the most variable element of the forelimb.

Here, we investigate turtle forelimb morphometrics and their relationship to habitat type using phylogenetic methods and linear discriminant analysis. We show that habitat classification accuracy increases considerably by including intramanual proportions (digit III metacarpal + non‐ungual phalanges + ungual). We then apply this method to the classification of fossil stem‐turtles and nanhsiungchelyids, for which paleohabitat reconstruction has been contentious.

## 2 MATERIALS AND METHODS

### 2.1 Dataset

To assess habitat classification accuracy using forelimb and manus proportions, it was necessary to first assemble a database of the relevant measurements. Joyce and Gauthier (2004) provided gross forelimb measurements (humerus + ulna + combined digit III metacarpal and phalanges lengths) in an appendix, which covered most turtle habitats and clades (n = 77 individuals) and which we used here. We supplemented these with our own measurements, including the lengths of the metacarpal, non‐ungual phalanges, and ungual of digit III (which forms the axis) of the manus (n = 56 individuals). We preferentially gathered these data from skeletonized museum specimens, but when this was not possible, we used x‐ray technology (Vidisco FlashX Pro Digital Radiography System), including scale, on fixed specimens to derive measurements for the bones of the manus. We were unable to measure museum specimens for *Hardella thurjii* and *Eremnochelys madagascariensis*, so instead we used CT data from Morphosource (https://www.morphosource.org). It was not possible to measure the same specimens as Joyce and Gauthier (2004), and so to combine our datasets, we scaled our measurements for digit III of the manus to the total manus length (similarly measured along digit III) of Joyce and Gauthier (2004). We measured only adult individuals following the methods of Joyce and Gauthier (2004), so it is unlikely that ontogenetic variation in relative manus length would affect our results. All raw measurements are provided on Dryad (Dudgeon et al., 2021).

### 2.2 Morphometrics

We performed three morphometric analyses: the first focusing on the major components of the forelimb from Joyce and Gauthier (2004) (humerus + ulna + manus), the second on the manus proper (digit III metacarpal + non‐ungual phalanges + ungual), and the third combining these two datasets (humerus + ulna, digit III metacarpal + non‐ungual phalanges + ungual). For each of these datasets, we log_10_‐transformed the measurements of the extant taxa and used phylogenetic generalized least squares (PGLS) regression to extract the residuals for subsequent analysis, which are corrected for both size and the phylogenetic nonindependence of taxa (see “Phylogentic trees” below for details on tree construction). We performed these PGLS regressions using different measurements as regressors to determine which produced the best fitting model. We also ran each regression under three different models of evolution: Brownian motion, Ornstein–Uhlenbeck, and lambda transformation. These PGLS regressions were run using the function “mvgls” from the R package *mvMORPH* v1.1.3 (Clavel et al., 2015), and model fit was evaluated using the Extended Information Criterion (EIC; Ishiguro et al., 1997) from the function “EIC” in the same R package.

The residuals from the best‐fitting evolutionary models from each regression were used in a linear discriminant analysis (LDA) to determine the accuracy of these measurements for predicting habitat. We classified habitat according to the six bins of Joyce and Gauthier (2004): “all bodies of water,” “moving or large bodies of water,” “primarily on land,” “primarily on land often in water,” “primarily on land seldom in water,” and “stagnant or small bodies of water.” Species classifications followed Joyce and Gauthier (2004), including *Kinixys* spp., which was re‐classified in another study as “primarily on land seldom in water” (Benson et al., 2011). We followed Joyce and Gauthier’s (2004) original classification for *Kinixys* spp. because recent observations have failed to find these turtles actively foraging in water, instead only finding that they can occur in proximity to water (*K*. *homeana*, Lawson, 2006; *K*. *belliana*, Demaya et al., 2020). We should emphasize that when following Benson et al.’s (2011) classification of *Kinixys* spp., the results did not significantly change (https://doi.org/10.5061/dryad.wwpzgmskn; Tables S1 and S2; Dudgeon et al., 2021). Species not appearing in the original study of Joyce and Gauthier (2004) were assigned habitats based on their entries in Ernst and Barbour (1989). The LDA was performed using the function “LDA” from the R package *MASS* v7.3.51.6 (Venables & Ripley, 2002). The classification accuracy for each LDA was calculated using a confusion matrix.

**FIGURE 1:**
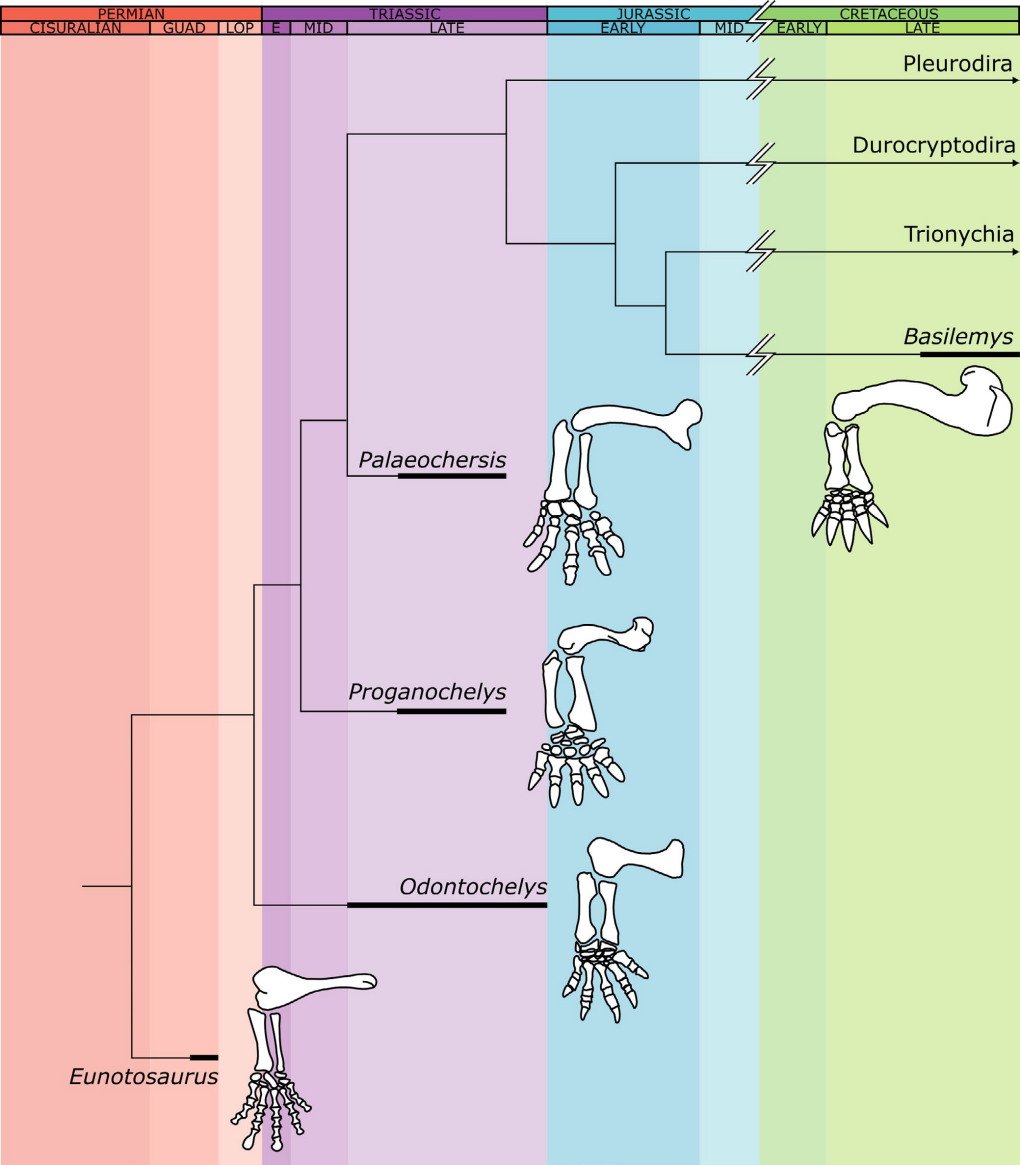
Time‐calibrated phylogeny of fossil turtles examined in this study (*Eunotososaurus africanus*, *Odontochelys semitestacea*, *Proganochelys talampayensis*, *Palaeochersis quenstedti*, *Basilemys variolosa*) relative to extant clades

We sought to determine the paleohabitats of five fossil species: the stem‐turtles *Eunotosaurus africanus*, *Odontochelys semitestacea*, *Proganochelys quenstedti*, and *Palaeochersis talampayensis*, and the nanhsiungchelyid *Basilemys variolosa* (a crown group turtle; Figure 1). To do this, we first log_10_‐transformed the fossil measurements and calculated their PGLS regression residuals and then used the best performing discriminant functions from each dataset (defined by classification accuracy) to predict their paleohabitats. These predictions were executed using the function “predict.lda” from the R package *MASS* (Venables & Ripley, 2002), with equally weighted prior probabilities. Visualizations of the major linear discriminant (LD) axes for each analysis were conducted using the function “ggplot” from the R package *ggplot2* v3.3.2 (Wickham, 2016), and “geom_mark_hull” from the R package *ggforce* v0.3.2 (Pederson, 2020).

### 2.3 Phylogenetic trees

The time‐calibrated phylogenetic trees of extant taxa used for the PGLS analyses came from Time Tree (timetree.org), a public database that compiles thousands of published phylogenies into a searchable, customizable time‐scaled tree. If species did not have a known entry in Time Tree, the most closely related species within the genus was used in its place. To append multiple specimens to the same species, which cannot be done within Time Tree itself, we manually inserted branches to the necessary lineages using the function “bind.tip” in the R package *phytools* v0.7.70 (Revell, 2012), with branch lengths set close to 0 (i.e., 0.1). Phylogenetic trees are available as supplementary information on Dryad (Dudgeon et al., 2021).

## 3 RESULTS

**FIGURE 2:**
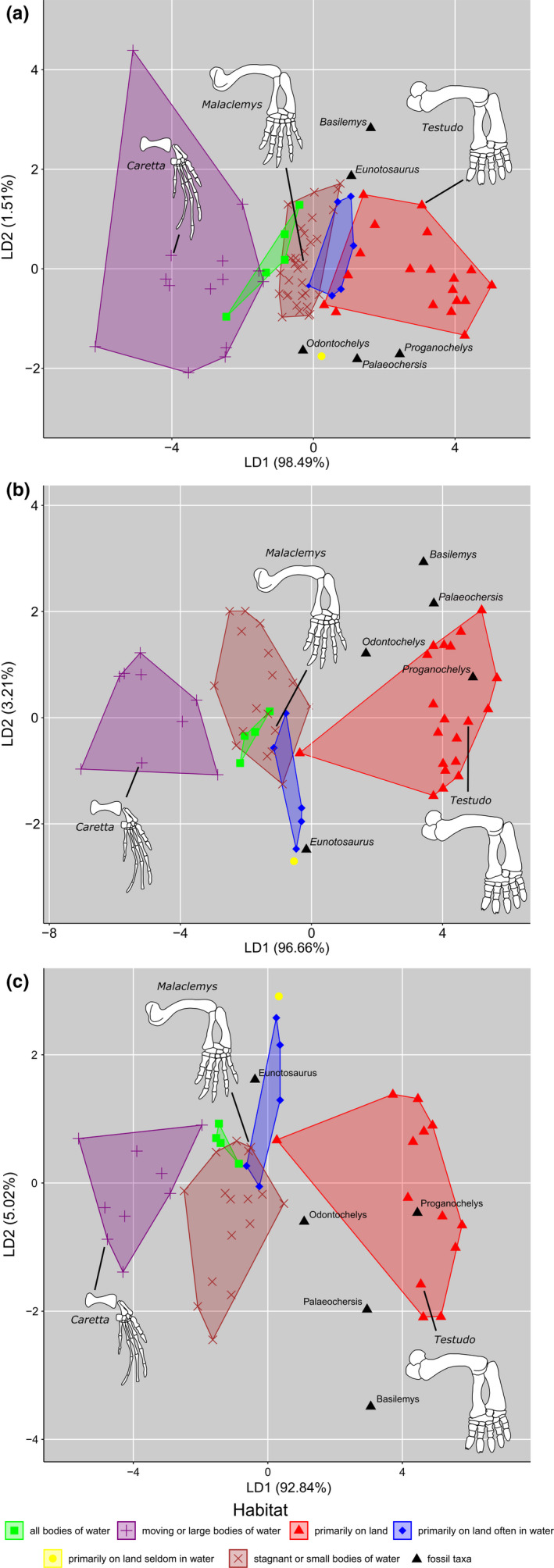
Plots of the extant habitat groups and fossil taxa on linear discriminant (LD) 1 vs. LD2. a, forelimb dataset; b, manual dataset; c, combined dataset

Across all PGLS models, the lambda transformations performed best, indicated consistently by the lowest EIC values (Table 1). We subjected the best performing evolutionary model for each regressor (lambda transformation) to LDA. The highest classification accuracies were consistently recovered across all regressors (83.33%) for the combined dataset (Table 1). The manual dataset had the next highest classification accuracies across regressors (best accuracy of 82.14% for the ulna regressor). The forelimb data of Joyce and Gauthier (2004) had the lowest classification accuracy of just 74.02% for the humerus and ulna regressors. Among the three manual measurements, the length of the non‐ungual phalanges best discriminated between ecological groups (LD1); shorter non‐ungual phalanges were more indicative of terrestriality, whereas longer non‐ungual phalanges were more indicative of aquatic locomotion (Figure 2B). Intraspecific (e.g., sexual) variation was typically expressed along LD2, reflecting differences in ungual length; males tend to have longer unguals and plot higher on the axis than females (e.g., *Trachemys scripta elegans*).

**TABLE 1:** Model results. Abbreviations: BM, Brownian motion; EIC, Extended Information Criterion; LDA, linear discriminant analysis; OU, Orenstein–Uhlenbeck; λ, lambda transformation

Dataset	Regressor	Model	EIC	log‐likelihood	test statistic	approx. F	*p*‐value	LDA accuracy (%)
Manus (metacarpals +non‐ungual phalanges +unguals)	Ulna	BM	295.55	−110.91	0.023	752	8.57*10^−43^	
		OU	−102.11	66.53	0.068	238.3	2.31*10^−30^	
		λ	−163.71	97.26	0.048	341.6	3.42*10^−34^	82.14
	Total length	BM	334.43	−140.57	0.065	249.4	7.67*10^−31^	
		OU	−33.13	33.61	0.20	69.28	3.58*10^−18^	
		λ	−75.73	56.85	0.19	74.18	8.61*10^−19^	78.57
Forelimb (humerus +ulna + manus)	Humerus	BM	−188.17	130.60	0.004	8502	3.64*10^−88^	
		OU	−247.38	151.70	0.006	5852	3.41*10^−82^	
		λ	−423.30	223.64	0.015	2408	4.54*10^−68^	74.02
	Ulna	BM	−204.38	128.87	0.008	4289	3.08*10^−77^	
		OU	−238.10	149.97	0.009	3974	5.04*10^−76^	
		λ	−424.34	223.51	0.021	1713	1.09*10^−62^	74.02
	Total length	BM	−668.58	389.98	5.91*10(−5)	411967	3.10*10^−154^	
		OU	−654.06	395.38	0.0001	208894	1.80*10^−143^	
		λ	−850.84	475.92	0.001	22094	7.09*10^−108^	72.73
Combined (manus +forelimb)	Humerus	BM	306.85	−102.65	0.045	228.2	2.38*10^−28^	
		OU	−241.34	142.62	0.024	432.4	4.13*10^−34^	
		λ	−253.01	157.82	0.031	330.3	1.15*10^−31^	83.33
	Ulna	BM	306.36	−100.22	0.035	295.5	1.10*10^−30^	
		OU	−47.89	66.57	0.051	200.7	3.26*10^−27^	
		λ	−246.42	155.40	0.038	269.5	7.77*10^−30^	83.33
	Total length	BM	265.56	−16.43	0.001	5834	1.61*10^−58^	
		OU	−468.23	269.26	0.003	2469	1.08*10^−50^	
		λ	−482.48	290.42	0.003	2884	4.17*10^(−52)	83.33

We then used the lambda transformation models with the highest classification accuracy for each dataset to predict the ecologies of the fossil taxa (Table 2). Because all three regressors for the combined dataset performed identically under a lambda transformation model, we used the ulna regressor for consistency with the other two datasets. Unsurprisingly, predictive likelihood generally increased with classification accuracy, although in very few of the tests did the fossil taxa plot among the extant forms. Under the two testable schemes, *Eunotosaurus africanus* was recovered as “primarily on land often in water.” *Odontochelys semitestacea* varied most widely in its habitat assignments, such that no two LDA predictions agreed; only the combined dataset (ulna regressor) and the forelimb dataset (ulna regressor) yielded a sufficiently confident habitat prediction of “all bodies of water” and “primarily on land seldom in water,” respectively. *Palaeochersis talampayensis* was variably recovered as having lived either “primarily on land” or “primarily on land seldom in water.” *Proganochelys quenstedtii* was consistently recovered as “primarily on land.” The nanhsiungchelyid *Basilemys variolosa* was recovered by the manual and combined models (the two best performing models) as “primarily on land,” with the forelimb model indicating “primarily on land often in water.” The datasets including intramanual proportions are more likely to return a more terrestrial signal for the included fossil taxa (Table 2).

**TABLE 2:** Predicted habitat assignments and posterior probabilities (in brackets) for the fossil taxa in each dataset (assuming lambda transformation models)

Taxon	Manual dataset (ulna regressor)	Forelimb dataset (ulna regressor)	Combined dataset (ulna regressor)
*Eunotosaurus africanus*	Primarily on land seldom in water (80.22%)	Primarily on land often in water (65.51%)	Primarily on land often in water (88.98%)
*Odontochelys semitestacea*	Primarily on land often in water (44.06%)	Primarily on land seldom in water (73.55%)	All bodies of water (82.04%)
*Palaeochersis talampayensis*	Primarily on land (99.93%)	Primarily on land seldom in water (83.43%)	Primarily on land (95.51%)
*Proganochelys quenstedti*	Primarily on land (99.99%)	Primarily on land (88.31%)	Primarily on land (99.99%)
*Basilemys variolosa*	Primarily on land (99.78%)	Primarily on land often in water (73.77%)	Primarily on land (99.37%)

## 4 DISCUSSION

Part of the reason for the many differing interpretations of fossil turtle habitats is that different proxies have led to inconsistent conclusions about ancestral habitat use. These have varied from shell proportions, bone isotopes, skull and middle ear morphology, osteohistology, and limb proportions (Benson et al., 2011; Dziomber et al., 2020; Foth et al., 2017, 2019; Joyce & Gauthier, 2004; Lichtig & Lucas, 2017; Rivera, 2008; Scheyer & Sander, 2007). We therefore anticipate the (fair) question: Why do we need another one?

As noted in the Introduction, many of these previously established proxies have proved to be poor predictors of habitat type. For example, although shell dimensions (e.g., carapace height, plastron width) are traditionally thought to reflect habitat type (e.g., aquatic vs. terrestrial environments), phylogeny is now thought to better predict shell dimensions than environment (Dziomber et al., 2020). Use of the forelimb proportions advocated by Joyce and Gauthier (2004) more reliably predict habitat than shell shape, but these, too, are still more strongly associated with clade membership (Benson et al., 2011).

Our new model, incorporating proportions of the manus (digit III metacarpal + non‐ungual phalanges + ungual), performs better than any of the traditional morphometric predictors. Compared to the performance of Joyce and Gauthier’s (2004) forelimb data, manual proportions have a 5–10% higher classification accuracy (Table 1). Classification accuracy may be increased further by combining measurements of the forelimb with those of the manus, but the improvement is comparably minor (1–5%). Where possible, intramanual proportions should be used to predict habitat, because this metric performs better than gross forelimb proportions alone.

Given the improved classification accuracies of those analyses incorporating manual data, we favor their habitat imputations for the fossil taxa we examined. As such, we find ourselves in agreement with recent authors who espouse a primarily terrestrial habitat for stem‐turtles (Joyce, 2015; Lichtig & Lucas, 2021; Lyson & Bever, 2020). A fossorial lifestyle has even been proposed for the earliest known stem‐turtle, *Eunotosaurus africanus* (middle Permian), which would account for its short, stiffened trunk and powerful forelimbs (Lyson et al., 2016). Our analysis identifies *E*. *africanus* as also having spent considerable time in water (Table 2). However, as first suggested by Joyce (2015) for *Odontochelys semitestacea*, the subtle lengthening of the manus—typically associated with aquatic habits—may instead reflect plesiomorphic hyperphalangy, rather than habitat adaptation. Our habitat assignments for *O*. *semitestacea* are highly variable but usually associated with aquatic environments (Table 2). Under no scenario is the genus recovered as of primarily marine origin, even though its fossils are derived from such deposits in China (Li et al., 2008).

We similarly recover the crown turtle *Basilemys variolosa* as having lived primarily on land (Table 2). The paleohabitats occupied by these and other nanhsiungchelyids have proved contentious over the years (Mallon & Brinkman, 2018), and the question of their habitat type inspired the present research. Many authors have argued for a strictly terrestrial habit for the nanhsiungchelyids, citing their stout limb proportions (Mlynarski, 1972; Tong & Li, 2019; Yeh, 1966) and associated armor ossicles (Hutchison and Archibald, 1986), well‐developed plastra (Lichtig & Lucas, 2017), and complex triturating surfaces of the jaws, suitable for rending tough, terrestrial browse (Brinkman, 1998). By contrast, others have cited the mobile nature of the humerus and the flattened shell as evidence for aquatic habits (Sukhanov, 2000; Sukhanov & Narmandakh, 1975). Nessov (1981) characterized these turtles as specialized for bottom‐walking in strong currents. Although we did not consider such forms of locomotion in our classification scheme, the chelydrids *Chelydra serpentina* and *Macrochelys temminckii* are notable extant bottom‐walkers (Willey & Blob, 2004), and these clearly cluster with the aquatic forms (Figure 2). We harbor no illusions to have settled the matter of nanhsiungchelyid habits but favor the terrestrial hypothesis in light of the mounting evidence for it.

Despite the strengths of our approach, we also recognize its limitations. Phalanges are small and prone to washing away, particularly in mid‐ to high‐energy depositional environments, which fossil turtles frequently inhabited (e.g., rivers and streams) (Voorhies, 1969). Therefore, getting good manual data from such depositional environments might be problematic, particularly compared to shell data. Certain habitat types (e.g., “primarily on land seldom in water,” “all bodies of water”) are underrepresented in our dataset, as they were in that of Joyce and Gauthier (2004). This is partly a reflection of the low frequency with which extant turtles actually inhabit these places. Finally, in the PGLS analyses, our use of “total length” regressors performed well (i.e., low EIC values), yet may be problematic because the same terms are necessarily shared by both the abscissa and ordinate, which can potentially lead to spurious correlation. We do not consider this to be a serious source of error because use of other, independent regressors often yielded comparable results.

## 5 CONCLUSIONS

Although raw forelimb proportions (humerus + ulna + manus) provide useful primary information about the habits of extinct turtles, intramanual proportions (digit III metacarpal + non‐ungual phalanges + ungual)—either alone or in combination with forelimb proportions—provide still further insight, when available. Our proposed approach, combining phylogenetic regression and linear discriminant analysis, shows that intramanual proportions are less influenced by phylogeny than other morphological proxies and provides a quantitative means by which to gauge classification accuracy. Our use of these methods lends additional support to the terrestrial hypothesis for turtle origins and a primarily terrestrial mode of life for the problematic nanhsiungchelyids.

## CONFLICT OF INTEREST

We have no conflicts of interest to disclose.

## AUTHOR CONTRIBUTIONS

**Thomas William Dudgeon:** Data curation (equal); Formal analysis (lead); Methodology (lead); Writing‐original draft (equal); Writing‐review & editing (equal). **Marissa C. H. Livius:** Conceptualization (equal); Data curation (equal); Investigation (equal); Writing‐original draft (equal); Writing‐review & editing (equal). **Noel Alfonso:** Data curation (supporting); Writing‐review & editing (supporting). **Stéphanie Tessier:** Data curation (equal); Writing‐review & editing (equal). **Jordan C. Mallon:** Conceptualization (equal); Data curation (equal); Formal analysis (supporting); Funding acquisition (lead); Supervision (lead); Writing‐original draft (equal); Writing‐review & editing (equal).

## Data Availability

All fossil specimens used in this study are formally accessioned in permanent, accessible repositories. All measurement data and phylogenetic trees used in this study are provided on Dryad (https://doi.org/10.5061/dryad.wwpzgmskn), along with the R code to run the morphometric analyses.
